# Comparison of short-acting versus extended-release nifedipine: Effects on hemodynamics and sympathetic activity in patients with stable coronary artery disease

**DOI:** 10.1038/s41598-019-56890-1

**Published:** 2020-01-24

**Authors:** John D. Parker, Matthew D’ Iorio, John S. Floras, Corey B. Toal

**Affiliations:** 1 Department of Pharmacology and Toxicology, University of Toronto, Ontario, Canada; 2 Division of Cardiology, Department of Medicine Mount Sinai Hospital and The Lunenfeld-Tanenbaum Research Institute, University of Toronto, Ontario, Canada

**Keywords:** Cardiology, Hypertension

## Abstract

We investigated the impact of short-acting and extended release nifedipine on sympathetic activity using radiotracer methodology in patients with stable coronary artery disease in order to more accurately document the response of the sympathetic nervous system to different formulations of this dihydropyridine calcium channel antagonist. Participants were randomized to placebo, short-acting or extended release nifedipine for 7–10 days. On the final day, systemic blood pressure, cardiac filling pressures, cardiac output, plasma norepinephrine (NE) and total body NE spillover were measured at baseline (time 0) and repeated at intervals for 6 hours. There were no differences in baseline measures between groups. Following the morning dose of study medication there were no changes in hemodynamics or sympathetic activity in the placebo group. However, there was a significant fall in blood pressure and a significant increase in total body NE spillover in both nifedipine groups. Importantly, the increase in sympathetic activity in response to short-acting nifedipine began earlier (30 minutes) and was much greater than that observed in the extended release group, which occurred later (270 minutes). These findings confirm that sustained therapy with nifedipine is associated with activation of the sympathetic nervous system which is dependent on the pharmacokinetics of the formulation.

## Introduction

Calcium channel antagonists play an important role in the management of hypertension and angina. Since their introduction into clinical practice there has been interest in their pharmacokinetic and pharmacodynamics related to the different dihydropyridines and effects on the sympathetic nervous system^1^. A number of studies have examined the impact of dihydropyridine calcium channel antagonists on the sympathetic nervous system using a variety of methods including plasma and/or urinary norepinephrine (NE) concentrations^2–11^, heart rate variability^12–17^, microneurography^18–24^ and, in a few cases, radiotracer measures of total body NE spillover^14,25,26^. Following acute dosing, a number of studies documented activation of the sympathetic nervous system^7,27–29^ but others have not^2,25,30,31^. In the setting of chronic therapy with dyhydropyridine calcium antagonists, some^6,14,20,32^, but not all^2,7,15,18,31,33^ studies reported continued sympathetic activation. Some studies documented differential effects on the sympathetic nervous system responses in young versus older patients^22,34^. This subject area was recently the subject of a detailed review^35^. Of note, the majority of these studies were carried out in normal volunteers or patients with hypertension. Only a small number of studies focussed on patients with known coronary artery disease^36–38^.

Interest in the dihydropyridine calcium channel blockers for treating hypertension and angina is based on their potent vasodilatory activity. Interest in the autonomic responses to the dihydropyridine calcium channel antagonists stems from the known adverse effects of sympathetic activation in a number of disease states including heart failure and coronary artery disease. This interest was heightened after reports by Furberg and colleagues that therapy with calcium channel antagonists had adverse effects on clinical outcome in patients with coronary artery disease^39,40^. Although subsequent large scale clinical studies demonstrated the cardiovascular safety of these drugs^41,42^, there has been continued interest in their impact on sympathetic activity.

The majority of studies have employed plasma concentrations of NE to assess the response of the sympathetic system with mixed results. The fundamental limitation of this approach is that changes in NE clearance as well as its release can alter plasma concentration of the amine. As such, changing plasma NE concentrations are not reliable as measures of sympathetic activity. Therefore, in the current study we employed radiotracer NE kinetic methods to examine the impact of therapy with short-acting nifedipine capsule and extended release nifedipine GITS, as compared to placebo, on sympathetic activity in a cohort of patients with coronary artery disease all of who were receiving concomitant therapy with a beta-blocker.

## Methods

This was a single-centre study designed to compare the pharmacokinetic, pharmacodynamic and sympathetic nervous system responses to two different formulations of the dihydropyridine calcium antagonist nifedipine. This protocol was approved by the Ethical Review Committee for Human Experimentation of the University of Toronto (Protocol Reference Number 1640). Written informed consent was obtained in all cases. This research was carried out in accordance with relevant guidelines including the International Council for Harmonization and the Declaration of Helsinki.

### Study population

Study participants included 40 patients with known coronary artery disease. and a history of stable angina. None had experienced an acute coronary syndrome or revascularization procedure within 3 months of their participation in the study. All patients had preserved left ventricular systolic dysfunction with an ejection fraction ≥45 percent by two-dimensional echocardiography and none had a history of congestive heart failure.

All participants were taking either atenolol (25–50 mg daily) or metoprolol (25–75 mg twice daily). Patients previously treated with a calcium channel antagonist had those medications discontinued for at least 10 days prior to randomization. No patient was treated with long acting oral or transdermal nitrates. The use of sublingual nitroglycerin on an as needed basis was permitted but no patient received sublingual nitroglycerin on the day that study measurements were acquired.

Patients were randomized to receive drug in a blinded manner using a double-dummy technique for allocation to placebo (n = 9), short-acting nifedipine 10 mg, TID (n = 16) or extended release nifedipine Gastrointestinal Therapeutic System (GITS) 60 mg, OD (n = 15). These medications were continued for 7–10 days. On the final day of the study, participants were admitted to the Cardiovascular Clinical Research Laboratory at 0800 hours prior to their morning dose of study medication.

### Hemodynamic measurements

Study procedures were carried out without sedation. Instrumentation was as follows: (1) using a right femoral venous approach a Swan-Ganz catheter was placed in the pulmonary artery, and (2) an arterial line was place in the right femoral artery for monitoring of arterial pressure and blood sampling. The Fick method was used to estimate cardiac output. At each time point heart rate (determined from the electrocardiogram), right atrial, pulmonary artery and femoral artery pressure were recorded. For all pressure measurements an average of 15 cardiac cycles was determined and the results expressed as the mean value.

### NE spillover measurements

Total body sympathetic activity was estimated using the NE spillover technique using a bolus followed by a constant infusion of tritiated NE (L-[2,5,6-^3^H]NE; New England Nuclear, Boston, MA) as previously described by Esler *et al*. and prior reports from our laboratory^43,44^. NE spillover rates were calculated from the following equations:

$${\rm{Total}}\,{\rm{body}}\,{\rm{NE}}\,{\rm{spillover}}\,({\rm{nmol}}/{\rm{\min }})=\frac{[3{\rm{H}}]{\rm{NE}}\,{\rm{infusion}}\,{\rm{rate}}}{{\rm{Plasma}}\,{\rm{NE}}\,{\rm{specific}}\,{\rm{activity}}}$$

### Biochemical analysis

Plasma concentrations of NE were quantified using HPLC and tritium-labeled NE concentrations were determined using scintillation spectroscopy^43^. Active plasma renin, atrial natriuretic peptide and endothelin-1 concentrations were determined using radioimmunoassay. Plasma nifedipine concentrations were measured using gas chromatography^45^.

### Study protocol

Following the diagnostic heart catheterization and insertion of catheters for hemodynamic monitoring, the patient was taken from the catheterization laboratory to an adjacent monitoring area. Subsequently, hemodynamics and cardiac output were measured twice at baseline, 15 minutes apart to confirm stability. If these measurements all varied by less ≤10% control measurements (time 0) were then carried out. After control, hemodynamic and neurochemical measurements patients received their morning doses of double-blind study medication (short-acting nifedipine 10 mg, nifedipine GITS 60 mg or placebo). Subsequently hemodynamics and cardiac output were repeated every 30 minutes for the next 6 hours. Blood sampling for catecholamines and plasma nifedipine concentrations were carried out at control (time 0) and at 30, 60, 90, 120, 270, 300, 330 and 360 minutes post study medication. Plasma atrial natriuretic peptide, the concentration of active plasma renin and endothelin-1 were measured at control as well as 60, 180 and 300 minutes post study medication.

### Statistical analysis

Baseline characteristics were compared using a two-way analysis of variance (ANOVA) with treatment (placebo, short-acting nifedipine or nifedipine GITS) as factor for continuous variables while the Fisher’s exact test was used to compare categorical variables. Changes in hemodynamic, neurochemical and nifedipine concentrations within and between groups over time were assessed using a two-way repeated measures analysis of variance with treatment (placebo, short-acting nifedipine or nifedipine GITS) as factor. This analysis allowed us to determine whether there was a significant change in measured variables over time and whether this time effect varied by treatment group (placebo, short-acting nifedipine or nifedipine GITS). The relationship between plasma nifedipine concentrations, total body NE spillover and mean arterial blood pressure was assessed using simple linear regression. Statistical analyses were carried out in Prism 8, MacOS, version 8.2.1.

## Results

Baseline characteristics of the 3 groups can be found in Table 1. At time 0, there were no differences in hemodynamic variables between the 3 groups (Table 1). Baseline plasma NE values and total body NE spillover were similar in the 3 treatment groups. Finally, there were no significant baseline differences in plasma concentrations of atrial natriuretic peptide, endothelin or plasma renin (Table 1).

**Table 1:** Patient Characteristics and Baseline (time 0) measurements.

Parameter	Placebo (n = 9)	Short-acting Nifedipine (n = 16)	Nifedipine GITS (n = 15)
Age (yrs)	63 ± 3	62 ± 2	63 ± 2
Sex (Male/Female)	8/1	16/5	13/2
Hyperlipidemia (n = /percent)	5/56	8/50	7/47
Diabetes (n = /percent)	3/33	6/38	5/33
Hypertension (n = /percent)	5/56	4/25	5/33
Prior myocardial infarction (n = /percent)	1/11	3/19	5/33
Heart rate (bpm)	62 ± 2	60 ± 3	59 ± 1
Mean arterial blood pressure (mmHg)	100 ± 5	103 ± 3	98 ± 3
Right atrial mean pressure (mmHg)	8 ± 2	10 ± 1	10 ± 1
Mean pulmonary artery pressure (mmHg)	18 ± 2	19 ± 1	20 ± 1
Cardiac Output (l/min)	5.3 ± 0.3	5.3 ± 0.3	5.5 ± 0.1
SVR (dynes.sec.cm-5)	1432 ± 163	1462 ± 81	1346 ± 80
Plasma NE (pmol/ml)	1.0 ± 0.3	1.0 ± 0.1	1.4 ± 0.2
Total body NE spillover (pmol/ml)	1551 ± 515	1637 ± 249	2069 ± 228
Atrial Natriuretic Peptide (pg/ml)	118 ± 19	151 ± 21	169 ± 16
Endothelin (pg/ml)	1.2 ± 0.1	1.3 ± 0.1	1.2 ± 0.1
Plasma Renin Activity (ng/mL/hour)	13.5 ± 1.8	14.8 ± 2.9	11.3 ± 1.1

SVR, systemic vascular resistance; NE, norepinephrine.

**Figure 1:**
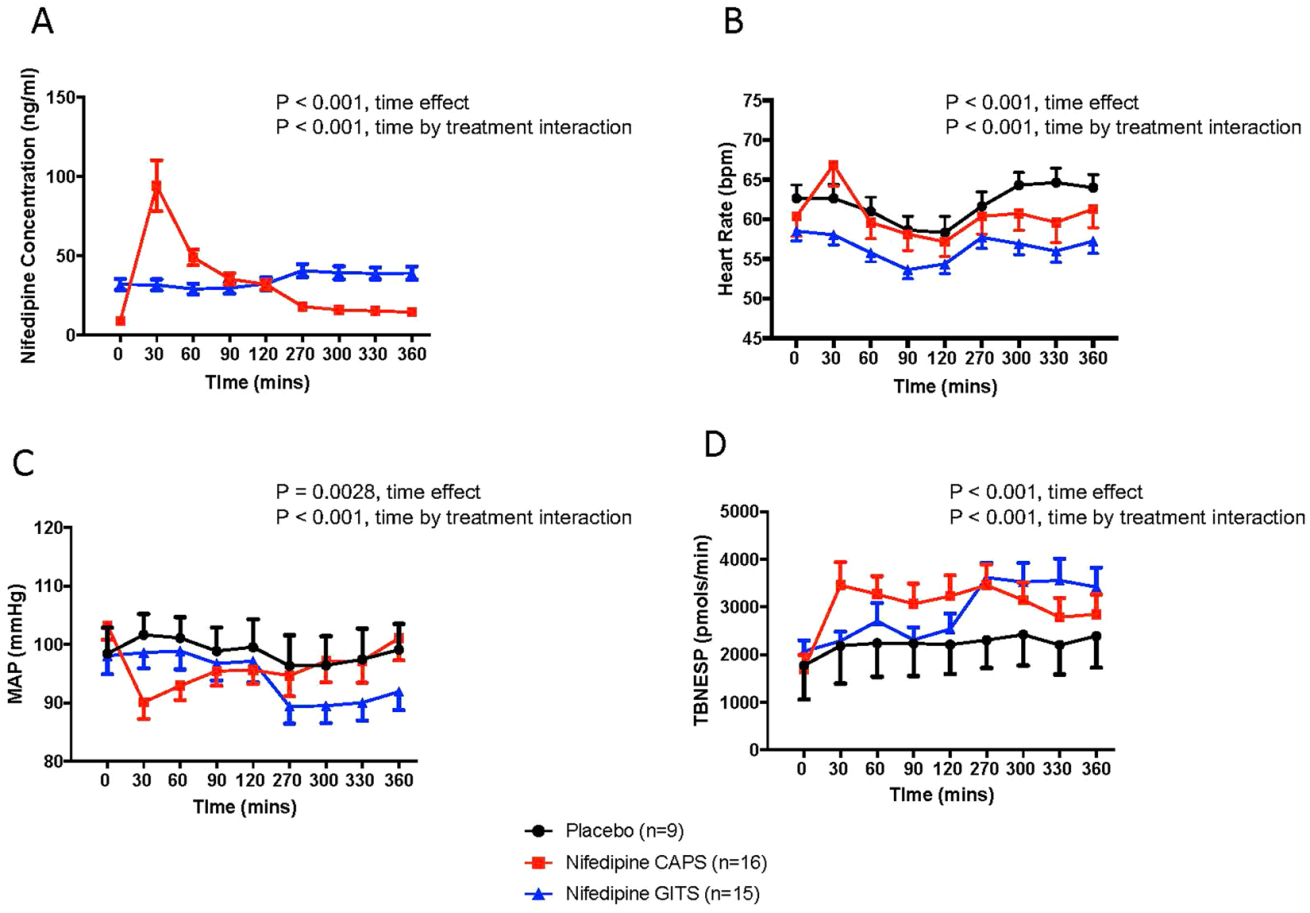
Panel A. Plasma Nifedipine concentrations in short-acting nifedipine and nifedipine GITS groups; Panel B. Heart rate responses in the 3 treatment groups; Panel C; Mean arterial blood pressure (MAP) responses in the 3 treatment groups; Panel D. Total norepinephrine spillover (TBNESP) responses in the 3 treatment groups. Each variable is presented before (time 0) and after administration of study medication (placebo, short-acting nifedipine or nifedipine GITS). In panel A (Nifedipine concentrations), the placebo group is not presented as nifedipine concentrations were not measurable). Time effect refers to whether there is a difference in the variable over time, without consideration of the treatment group. Time by treatment interaction refers to whether the time effect varies by treatment group (placebo, short-acting nifedipine or nifedipine GITS).

After administration of short-acting nifedipine there was a rapid increase in plasma nifedipine concentrations which peaked at the 30 minute time point and remained elevated for 120 minutes (P < 0.001, Fig. 1A). After administration of nifedipine GITS, nifedipine concentrations stayed relative constant during the first 120 minutes following drug adnministration but rose significantly at the 270 minute time point and remained elevated through the remainder of study measurement time points (P < 0.001, Fig. 1A). The ANOVA revealed a significant difference in the change in nifedipine concentrations over time between the 2 nifedipine groups (P < 0.001, Fig. 1A)

Following administration of study medication, the ANOVA revealed significant changes in heart rate and mean arterial blood pressure over time and a significant interaction with treatment allocation. There was no significant change in heart rate following administration of placebo (Fig. 1B). There was a rapid increase in heart rate following administration of short-acting nifedipine at 30 minutes (P < 0.001, Fig. 1B). This increase in heart rate rapidly returned to control values by the 60-minute time point. There was no significant change in heart rate following administration of nifedipine GITS (Fig. 1B). The ANOVA revealed a significant difference in the change in heart rate over time between the 3 treatment groups (P < 0.001, Fig. 1B). There was no change in mean arterial blood pressure over time in the placebo group (Fig. 1C). In both nifedipine groups, there was a significant fall in mean arterial blood pressure following study medication. This began at the 30-minute time point following short-acting nifedipine and remained significant through the 300-minute time point (P = 0.0028, Fig. 1C). Following administration of nifedipine GITS, the reduction in mean arterial blood pressure began at 270 minutes and persisted through the 360-minute time point (P = 0.0028, Fig. 1C). The ANOVA revealed a significant difference in the change in mean arterial blood pressure over time between the 3 treatment groups (P < 0.001, Fig. 1C).

There were also significant changes in cardiac output after administration of study medication. In the placebo group cardiac output did not change over time (Table 2). In the short-acting nifedipine group there was an increase in cardiac output at 30 minutes, although this variable returned to baseline at 60 minutes and remained unchanged thereafter (P < 0.001, Table 2). In the nifedipine GITS group there was a small but consistent reduction in cardiac output which began at 90 minutes and persisted until the end of the study period (P < 0.001, Table 2). The ANOVA revealed a significant difference in the change in cardiac output over time between the 3 treatment groups (P = 0.017, Table 2). There was no change in right atrial pressure or mean pulmonary artery pressure over time in any of the treatment groups (Table 2).

**Table 2:** Changes pre and post morning dose of study medication.

	Time 0	30 min	60 min	90 min	120 min	270 min	300 min	330 min	360 min	Time Effect	Time by Tx
**Placebo group**											
RAP (mmHg)	8 ± 1	8 ± 1	8 ± 1	8 ± 1	9 ± 1	9 ± 2	10 ± 1	9 ± 1	9 ± 1	NS	NS
PAM (mmHg)	19 ± 2	20 ± 2	21 ± 2	18 ± 2	21 ± 3	21 ± 3	21 ± 3	19 ± 3	19 ± 2	NS	NS
CO (L/min)	5.2 ± 0.3	5.3 ± 0.3	5.0 ± 0.2	5.0 ± 0.3	4.9 ± 0.3	4.8 ± 0.3	5.0 ± 0.4	4.8 ± 0.3	4.7 ± 0.3	NS	P = 0.017
NE (pmol/ml)	1.0 ± 0.3	1.2 ± 0.3	1.2 ± 0.3	1.3 ± 0.4	1.4 ± 0.3	1.4 ± 0.3	1.4 ± 0.3	1.2 ± 0.3	1.3 ± 0.3	NS	P < 0.0001
ANP (pg/ml)	118 ± 19	—	115 ± 13	—	—	—	119 ± 23	—	—	NS	NS
**Short-acting nifedipine group: Changes pre and post morning dose of study medication**											
RAP (mmHg)	10 ± 1	10 ± 1	10 ± 1	10 ± 1	10 ± 1	10 ± 1	11 ± 1	11 ± 1	11 ± 1	NS	NS
PAM (mmHg)	19 ± 1	20 ± 1	18 ± 1	18 ± 1	19 ± 1	18 ± 1	19 ± 1	19 ± 1	19 ± 1	NS	NS
CO (L/min)	5.3 ± 0.3	6.2 ± 0.3	5.3 ± 0.2	5.0 ± 0.2	5.0 ± 0.2	4.9 ± 0.2	4.7 ± 0.2	4.6 ± 0.3	4.8 ± 0.2	P < 0.0001	P = 0.017
NE (pmol/ml)	1.0 ± 0.1	1.8 ± 0.2	1.9 ± 0.2	2.0 ± 0.2	1.7 ± 0.1	1.7 ± 0.2	1.6 ± 0.2	1.5 ± 0.2	1.5 ± 0.2	P = 0.0018	P < 0.0001
ANP (pg/ml)	151 ± 21	—	151 ± 22	—	—	—	108 ± 10	—	—	P = 0.04	NS
**Nifedipine GITS group: Changes pre and post morning dose of study medication**											
RAP (mmHg)	10 ± 1	10 ± 1	10 ± 1	10 ± 1	10 ± 1	10 ± 1	9 ± 1	10 ± 1	10 ± 1	NS	NS
PAM (mmHg)	20 ± 1	20 ± 1	20 ± 1	20 ± 1	20 ± 1	20 ± 1	21 ± 1	20 ± 1	21 ± 1	NS	NS
CO (L/min)	5.5 ± 0.2	5.4 ± 0.2	5.1 ± 0.1	4.8 ± 0.2	4.8 ± 0.2	4.8 ± 0.2	4.8 ± 0.2	4.7 ± 0.2	4.7 ± 0.2	< 0.0001	P = 0.017
NE (pmol/ml)	1.4 ± 0.2	1.5 ± 0.2	1.5 ± 0.2	1.5 ± 0.2	1.6 ± 0.2	1.8 ± 0.2	1.8 ± 0.2	1.7 ± 0.2	1.7 ± 0.2	P = 0.02	P < 0.0001
ANP (pg/ml)	169 ± 16	—	144 ± 16	—	—	—	135 ± 21	—	—	P = 0.04	NS

RAP, right atrial pressure; PAM, mean pulmonary artery pressure, CO, cardiac output, NE, Norepinephrine; ANP, atrial natriuretic peptide. Time by Tx indicates wether there was a significant interaction between the time effect and treatment group.

In the placebo group there was an increase in plasma NE over time, but these changes were not significant (Table 2). In the short-acting nifedipine group there was an increase in plasma NE which began at 30 minutes and continued throughout the study period (P < 0.001, Table 2). In the nifedipine GITS group, plasma NE did increase, although this increase began at 270 minutes and continued until the end of the study period (P = 0.0005, Table 2). The ANOVA revealed that these changes over time varied as a function of the treatment group (P < 0.001, Table 2). There was no change in total body NE spillover in the placebo group (Fig. 1C). In both nifedipine groups, there was a significant increase in total body NE spillover following study medication. This began at the 30-minute time point following short-acting nifedipine and remained elevated through the 270-minute time point (P < 0.001 Fig. 1D). Following administration of nifedipine GITS, the increase in total body NE spillover began later, at the 270-minute time point and remained elevated through the 360-minute time point (P < 0.001, Fig. 1C). The ANOVA revealed a significant difference in the time course of changes in total body NE spillover between the 3 treatment groups (P < 0.001, Fig. 1C).

**Figure 2:**
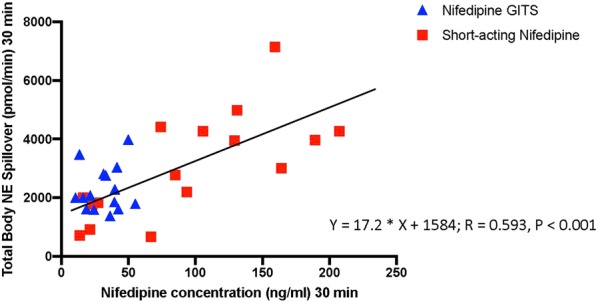
Linear regression, nifedipine concentrations vs total norepinephrine spillover (TBNESP) at the 30-minute time point. Data includes nifedipine concentrations in both the short-acting nifedipine and nifedipine GITS groups. The placebo group is not included as nifedipine concentrations were not measurable in this group.

There was a significant positive correlation between the plasma concentration of nifedipine and body NE spllover from time 0 to 30 minutes (Y = 17.2 * X + 1584; R = 0.593, P < 0.001, Fig. 2). There was also a significant positive correlation between the plasma concentration of nifedipine and total body NE spillover when all time points in both active treatment groups were combined (Y = 13.99 * X + 2482; R = 0.224, P < 0.001). There was also a significant negative correlation between the change in plasma concentration of nifedipine and the change in mean arterial blood pressure from time 0 to 30 minutes (Y = −0.09554 * X − 1.176; R = 0.798, P < 0.001).

There was a significant decrease in atrial natriuretic peptide in both the short-acting nifedipine and nifedipine GITS groups (P = 0.04, Table 2) but the ANOVA revealed that this difference did not vary by treatment group. There was no change in the concentration of active plasma renin or endothelin-1 following study medication in any of the treatment groups (Table 2).

## Discussion

This study documents that in patients with stable coronary artery disease, daily therapy with nifedipine, in both a short-acting as well as in an extended release (GITS) formulation, is associated with sympathetic activation as compared to those treated with placebo. At baseline, following 7–10 days of therapy, total body NE spillover was similar in the 3 treatment groups. However, total body NE spillover rose immediately after the subsequent dose of short-acting nifedipine and this increase persisted for the next 6 hours. In contrast, after the morning dose of nifedipine GITS, total body NE spillover increased, although this increase did not begin until 3–4 hours following subsequent dosing. As such, this study demonstrates that both short-acting and extended release formulation of nifedipine GITS are associated with increases in total body NE spillover, the time course of which is dependent upon their individual pharmacokinetic and pharmacodynamic characteristics. Importantly, the magnitude of sympathetic activation in response to short-acting nifedipine is greater than that observed in the extended release preparation reflecting the very abrupt fall in blood pressure caused by the short-acting preparation.

Prior studies examining the impact of therapy with nifedipine GITS on measures of the sympathetic nervous system have yielded contradictory results. Many of these studies involved patients with hypertension and there were significant differences in terms of study design, formulation of drug, dose and measures of sympathetic activity. Most of these studies used plasma NE as a marker for changes in sympathetic activity. Some demonstrated the plasma NE was increased during acute and/or sustained therapy with nifedipine GITS^4,5,15,24,46^, although others reported no change in this variable^2,22,47^. Some studies used measurement of peripheral muscle sympathetic nerve activity as a more direct measure of sympathetic responses. Some of these studies demonstrated an increase in muscle sympathetic activity in response to nifedipine^19,24^ although no change was observed on other reports^18^. These discordant results likely reflect differences in dose, formulation and study design.

Importantly, this is the first report using radiotracer methodology to examine the impact of nifedipine on the activity of the sympathetic nervous system. This approach allows for measurement of total body NE spillover which is an integrated summation of sympathetic activity in all vascular beds. This approach provides a measurement which accounts for both changes in production and clearance of NE. As such, it provides a more specific estimate of changes in sympathetic activity than measures of plasma NE, the concentration of which can be changed by both changes in its production and clearance. Indeed, we have previously demonstrated that changes in NE clearance are directly proportional to changes in cardiac output and that plasma NE can remain unchanged despite an increase in total body NE spillover in situations where there is a concurrent increase in NE clearance^43^.

As mentioned above, some studies with various formulations of nifedipine have reported different effects of nifedipine on muscle sympathetic nerve activity^18,19,24^. Although the microneurographic technique does provide a direct measure of efferent nerve traffic to skeletal muscle, sympathetic activity to this vascular bed may not be representative of changes in sympathetic activity to other vascular beds. Further, measures of efferent post-ganglionic firing frequency cannot anticipate the prejunctional regulation of NE release from sympathetic nerve terminals.

The current observations were made in patients with stable coronary artery disease with well-preserved left ventricular systolic function and no history of congestive heart failure. There have been only limited prior reports of the sympathetic responses to nifedipine in patients with stable coronary artery disease. These studies used plasma NE as a marker of sympathetic responses with, as in studies with hypertension, inconsistent results^36–38^. To date, our observations represent the most comprehensive assessment of the effects of nifedipine in both short-acting and extended release formulations in patients with stable coronary artery disease. However, it is important to emphasize that patients in the current study were all receiving therapy with a beta-1 selective beta-blocker (either atenolol or metoprolol). In prior reports of the effects of nifedipine on sympathetic nervous system responses other vasoactive medications, including beta-blockers were either not given or withdrawn prior to study measurements. It is possible that ongoing adrenergic receptor blockade may explain the sympathetic activation observed in the current study. For example, by limiting the heart rate response to the vasodilator effect of nifedipine, ongoing beta-blockade could have enhanced arterial baroreceptor mediated increases in sympathetic responses. Nevertheless, since most patients with coronary artery disease receive beta-blockers, our observations are representative of the real-world use of nifedipine in this population. This was the case in the ACTION study where nifedipine GITS was shown to be safe in patients with stable coronary artery disease. In that study, 80 percent of patients were treated with concomitant beta-blockers^41^.

There was a significant positive correlation between the change in plasma nifedipine concentration and the change in total body sympathetic activity. This almost certainly results from the effect of increasing plasma concentrations on systemic arterial blood pressure. In fact, there was a highly significant negative correlation between increasing plasma concentrations of nifedipine and the resulting decrease in systemic arterial blood pressure. Of note, most of these changes were mediated by the large changes in plasma nifedipine concentrations that occurred following the administration of the short-acting formulation of nifedipine. Nevertheless, there was a significant correlation between absolute nifedipine concentrations (when data from both formulations was combined at all time points) and total body sympathetic activity. These observations serve as an explanation for the observation that in the extended release nifedipine group, the decrease in blood pressure and an increase in total body NE spillover occurred at 270 minutes. These changes occurred in parallel with an increase in plasma nifedipine concentration that occurred over the same time period. Therefore, it appears that both the hemodynamic and neurochemical responses to nifedipine are closely linked to changes to changes in plasma concentrations of nifedipine.

Therefore, this study demonstrates that daily therapy with nifedipine, in both short-acting and extended release formulations is associated with evidence of increased generalized activity of the sympathetic nervous system as assessed the radiotracer NE kinetics. The time course of this activation varies in a manner which is consistent with the pharmacokinetics of the respective formulations. The clinical significance of these findings is unclear, as long-term outcome studies have documented the safety of extended release nifedipine in patients with both hypertension and chronic coronary artery disease. It should be noted that in the ACTION study, which demonstrated the safety of Nifedipine GITS in patients with stable coronary artery disease, 80 percent of enrolled patients were taking a beta-adrenergic blocker. Given the potential adverse effects of sustained increases in sympathetic activity in patients with cardiovascular disease, the current study suggests that when patients with coronary artery disease are treated with nifedipine, concurrent therapy with a beta-adrenergic blocking agent should be used whenever possible. Furthermore, less than 50 percent of these patients had concomitant hypertension and it is possible that the sympathetic responses to nifedipine would be different in a cohort of patients all of whom had hypertension^41,42^. Finally, our study confirms that the use of short-acting nifedipine is associated with abrupt decreases in systemic arterial blood pressure and greater reflex increases in activity of the sympathetic nervous system which serve to emphasize that short-acting formulations of dihydropyridine antagonists should be avoided in patients with cardiovascular disease.

The study protocol and data presented in this manuscript are available upon request from the corresponding author.
